# Supplementary material for: Novel Driver Strength Index highlights important cancer genes in TCGA PanCanAtlas patients
Source: PeerJ. 2022 Aug 11;10:e13860. doi: 10.7717/peerj.13860 (PMC9375969; doi:10.7717/peerj.13860)
Supplement: Supplemental Information 2 [file peerj-10-13860-s002.zip › COHORTS/genes plots/cohorts/2021_8_16_14_9_distribution_cohorts_females_PCPG.pdf]

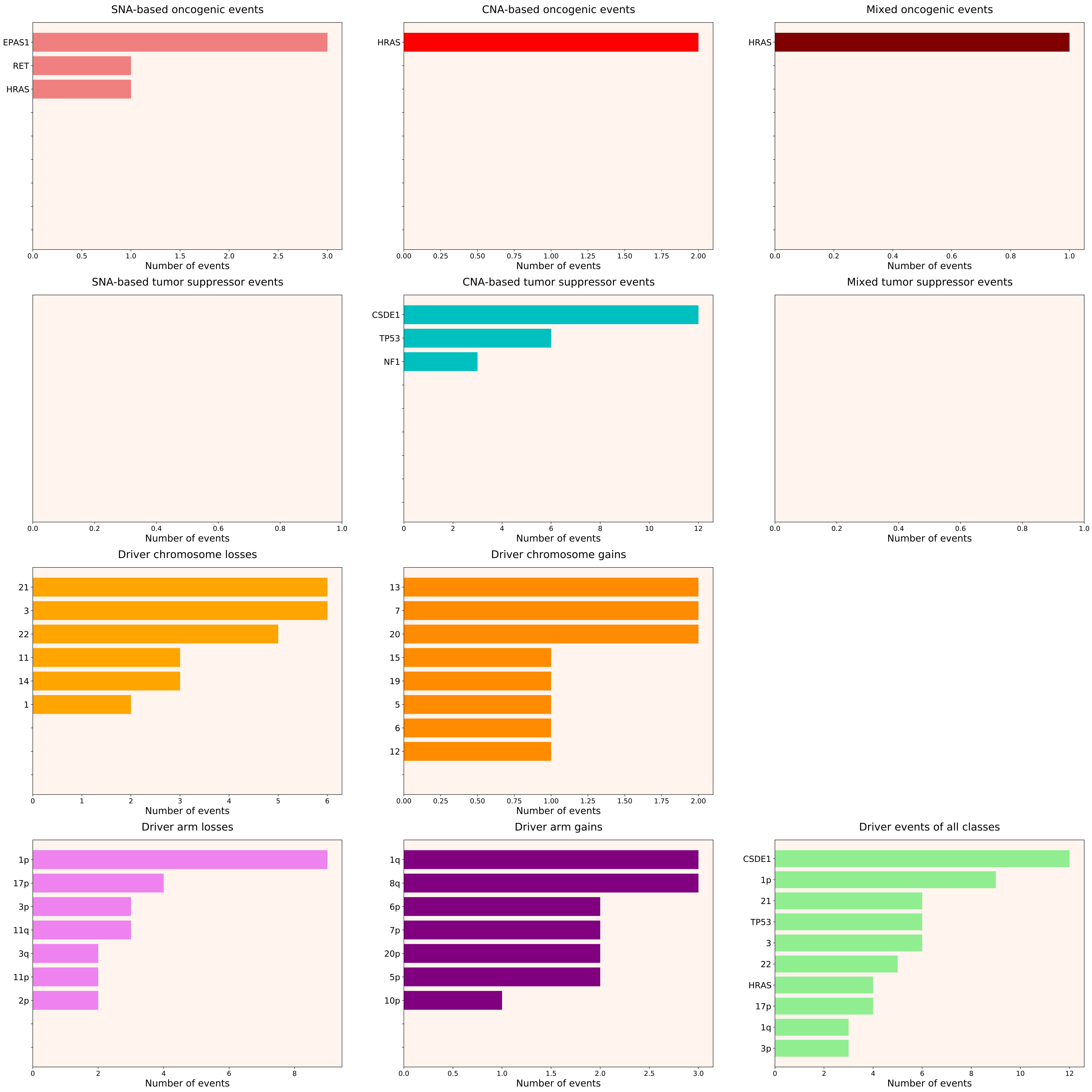

SNA-based oncogenic events
EPAS1
RET
HRAS
Number of events
CNA-based oncogenic events
HRAS
Number of events
Mixed oncogenic events
HRAS
Number of events
SNA-based tumor suppressor events
Number of events
CNA-based tumor suppressor events
CSDE1
TP53
NF1
Number of events
Mixed tumor suppressor events
Number of events
Driver chromosome losses
21
3
22
11
14
1
Number of events
Driver chromosome gains
13
7
20
15
19
5
6
12
Number of events
Driver arm losses
1p
17p
3p
11q
3q
11p
2p
Number of events
Driver arm gains
1q
8q
6p
7p
20p
5p
10p
Number of events
Driver events of all classes
CSDE1
1p
21
TP53
3
22
HRAS
17p
1q
3p
Number of events
